# Supplementary material for: Temporal Effects of Quercetin on Tight Junction Barrier Properties and Claudin Expression and Localization in MDCK II Cells
Source: Int J Mol Sci. 2019 Oct 2;20(19):4889. doi: 10.3390/ijms20194889 (PMC6801663; doi:10.3390/ijms20194889)
Supplement: Supplementary file 1 [file ijms-20-04889-s001.pdf]

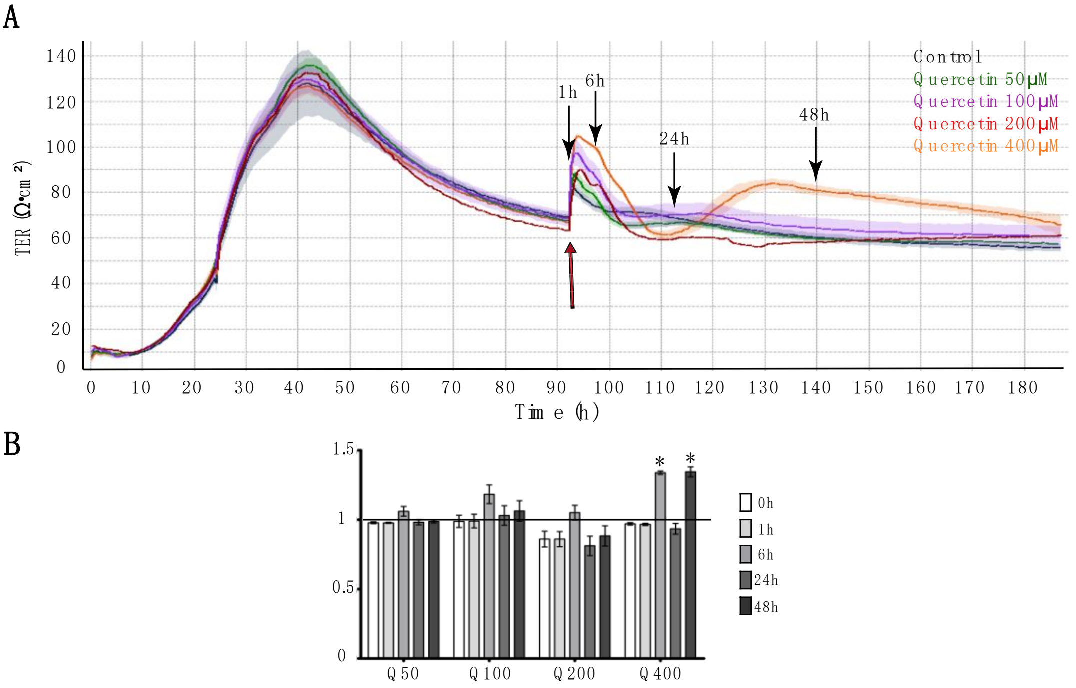

Figure S1. Quercetin dose response on TER. **(A)** Comparison of TER of control (black) versus cells treated with 50μM (green), 100μM (purple), 200μM (red) or 400μM of quercetin (orange). **(B)** Measurement of quercetin treated TER values relative to control cells at different time points and different treatment concentrations. The horizontal line delineates control level. *P<0.05.

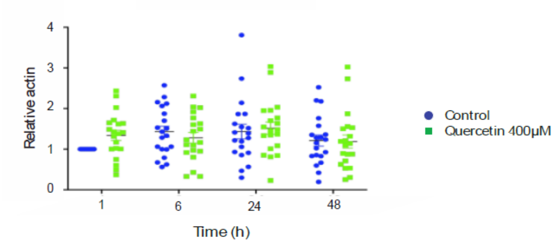

Densitometry of all actin bands used for normalization for control and quercetin treated cells. Each band is normalized to control actin at 1h for each of the blots. Two-way Anova and Sidak´s multiple comparison test was performed and no significance was observed (P_{int}=0.32; P_{time}=0.097; P_{treat}=0.52). Mean and SEM are plotted. Each dot corresponds to an independent experiment.
